# 4.K. Workshop: Promoting and enhancing health literacy through school interventions

**DOI:** 10.1093/eurpub/ckac129.246

**Published:** 2022-10-25

**Authors:** Organised by: EUPHA Working Group Health Literacy, EUPHA-HP, EUPHA-CAPH, Technical University Munich, Chair persons: Orkan Okan (EUPHA-HP), Julia Dratva (EUPHA-CAPH)

## Abstract

The promotion and enhancement of health literacy in school-aged children is an important public health and education goal. To ensure healthy behaviours and attitudes in adulthood, children and adolescents have to learn about health and well-being early in life. Toward this goal, schools are considered the most important setting to address health literacy. Interventions delivered in schools can reach all children regardless of their social, cultural or development background. In the education sector, health literacy interventions can address not only the skills, attitudes and knowledge of students, but that of teachers, principals, health and education staff, as well as the whole school setting. Health literacy is linked to education, while it mainly addresses how people manage health information. In this context, health literacy enables children and adolescent to find, understand and critically appraise health information and use it to guide informed health decisions and promote health behaviour. While this understanding targets personal competencies of individuals, health literacy is also directed towards the environment. For example, the concept of organizational health literacy aims at increasing health literacy capacities of the environment and within settings, such as schools. This makes health literacy an important concept at the intersection of behaviour change (agency: personal health literacy) and social change (structure: organizational health literacy). While the behavioural approach is most promising to equip students with the necessary skills to act as their own agents of health information management and improve their health agency capacities, the social approach further includes consideration of social determinants of health to provide child- and adolescent-friendly environments and conditions. Therefore, effective health literacy interventions should aim at combining behavioural and social approaches, especially in such vulnerable groups as children and adolescents. The purpose of this workshop is to present and discuss four interventions on health literacy drawing on behavioural and social sciences. (i) The first presentation will introduce the Health Literacy Toolbox for Schools (Tool-HLCA). (ii) The second presentation will introduce a school curriculum for classroom based Mental Health Literacy (HLCA-IMPRES). (iii) The third presentation will provide the organizational health literacy school intervention: Health Literate Schools (HeLit-Schools). (iv) The fourth will present applied data on mental health literacy in teachers and an instrument to measure action competencies of teachers with respect to promoting their students’ mental health (MHAC). Each project will be given ten minutes to present their interventions and main findings, including time to discuss with the audiences and get feedback. The participants in turn will learn about recent empirical findings they can use in health literacy practice and policy.

**Key messages:**

• Children and adolescent will benefit from early interventions to develop healthy behaviours and lifestyles in their life-course.

• Health literacy is an important school topic and should be addressed within behavioural and social approaches.

